# Hunting behavior and feeding ecology of Mojave rattlesnakes (*Crotalus scutulatus*), prairie rattlesnakes (*Crotalus viridis*), and their hybrids in southwestern New Mexico

**DOI:** 10.1002/ece3.10683

**Published:** 2023-11-07

**Authors:** Dylan W. Maag, Yannick Z. Francioli, Noelle Shaw, Ashana Y. Soni, Todd A. Castoe, Gordon W. Schuett, Rulon W. Clark

**Affiliations:** ^1^ Department of Biology San Diego State University San Diego California USA; ^2^ Department of Evolution, Ecology, and Organismal Biology University of California, Riverside Riverside California USA; ^3^ Department of Biology University of Texas at Arlington Arlington Texas USA; ^4^ Department of Biology, Neuroscience Institute Georgia State University Atlanta Georgia USA; ^5^ Chiricahua Desert Museum Rodeo New Mexico USA

**Keywords:** ambush predation, body condition, foraging, hybridization, pitvipers, Reptilia

## Abstract

Predators must contend with numerous challenges to successfully find and subjugate prey. Complex traits related to hunting are partially controlled by a large number of co‐evolved genes, which may be disrupted in hybrids. Accordingly, research on the feeding ecology of animals in hybrid zones has shown that hybrids sometimes exhibit transgressive or novel behaviors, yet for many taxa, empirical studies of predation and diet across hybrid zones are lacking. We undertook the first such field study for a hybrid zone between two snake species, the Mojave rattlesnake (*Crotalus scutulatus*) and the prairie rattlesnake (*Crotalus viridis*). Specifically, we leveraged established field methods to quantify the hunting behaviors of animals, their prey communities, and the diet of individuals across the hybrid zone in southwestern New Mexico, USA. We found that, even though hybrids had significantly lower body condition indices than snakes from either parental group, hybrids were generally similar to non‐hybrids in hunting behavior, prey encounter rates, and predatory attack and success. We also found that, compared to *C. scutulatus*, *C. viridis* was significantly more active while hunting at night and abandoned ambush sites earlier in the morning, and hybrids tended to be more *viridis*‐like in this respect. Prey availability was similar across the study sites, including within the hybrid zone, with kangaroo rats (*Dipodomys* spp.) as the most common small mammal, both in habitat surveys and the frequency of encounters with hunting rattlesnakes. Analysis of prey remains in stomachs and feces also showed broad similarity in diets, with all snakes preying primarily on small mammals and secondarily on lizards. Taken together, our results suggest that the significantly lower body condition of hybrids does not appear to be driven by differences in their hunting behavior or diet and may instead relate to metabolic efficiency or other physiological traits we have not yet identified.

## Graphical abstract

Complex traits, such as predatory behaviors, are partially controlled by many co‐evolved gene complexes, which can be disrupted by interspecific hybridization. We quantified the hunting behavior, prey resource base, and diet of snakes dispersed across a hybrid zone between Mojave rattlesnakes (*Crotalus scutulatus*) and prairie rattlesnakes (*Crotalus viridis*). Generally, even though hybrids were in poorer body condition than snakes from either parental lineage, all snakes displayed similar hunting behaviors, diet, and rates of predatory success.

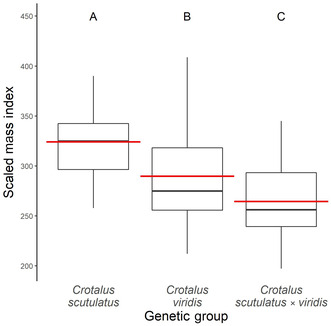

## 1 INTRODUCTION

All organisms need to assimilate energy from the environment to survive and reproduce. For predators, their success in acquiring energy depends directly on their ability to locate, subdue, and consume other animals. Anatomical, physiological, and behavioral traits related to these processes are consistent targets of natural selection due to their impacts on a predator's growth, development, and fecundity (reviewed in Apicella, 2014; Feldman et al., 2009; Holding et al., 2021; Schoener, 1971). The hunting behavior of predators also must account for complex ecological interactions. For example, for predators to hunt effectively, they must not only interact with their prey but also compete for resources with conspecifics and other species and contend with a range of obstacles imposed by the abiotic environment (e.g., temperature, light levels, geological structures, terrestrial vs. aquatic, etc.). Because of their close association with fitness, behavioral traits associated with foraging behaviors and diet can have an important role in shaping reproductive isolation between closely related lineages (Good et al., 2000; Grant & Grant, 1996; Peters & Kleindorfer, 2015).

Studying how traits related to predation and foraging are expressed across hybrid zones can provide valuable opportunities to understand the ramifications of disrupting co‐evolved phenotypes. Past studies on hybrid zones have identified important links between feeding ecology and hybridization dynamics. When parental lineages have similar hunting or foraging traits, hybrids often possess phenotypes that are similar to one parent or have traits of both parental phenotypes (Peters & Kleindorfer, 2015; Sas et al., 2005; Vamosi et al., 2000). Hybrids may also be intermediate between parentals on average, but much more variable, as increased variation in phenotype is commonly found across hybrid zones (Barton, 2001; Mallet, 2007; Rieseberg et al., 2007). Thus, hybrids can express more novel or extreme (transgressive) traits when compared to parental populations (Harrison & Larson, 2014; Rieseberg et al., 1999; Stelkens et al., 2009). When transgressive hybrid traits allow hybrids to fill empty niches, subsequent adaptive evolution can lead to transgressive segregation (Seehausen, 2004). For example, hybrid cichlids were found to be more efficient than parentals at exploiting novel food types, but less efficient with food types that were routinely encountered by either parental lineage (Selz & Seehausen, 2019). In a study on piscivorous birds, hybrid gulls (*Larus galucescens* × *L. occidentalis*) had significantly more fish in their diet than parental individuals (Good et al., 2000). Furthermore, the high fish diet of hybrids was associated with increased growth and survival of chicks, ultimately leading to higher reproductive success for hybrids when compared to the parental gulls (Good et al., 2000). Though data are few, it is possible that traits associated with feeding may represent extrinsic factors (i.e., factors related to ecological or environmental conditions extrinsic to phenotype) that can impact hybridization in other vertebrate systems.

The prevalence of hybridization in several lineages of pitvipers (Serpentes: Crotalinae) represents a unique opportunity to explore the relationship between feeding ecology and hybridization dynamics in snakes (Bailey, 1942; Campbell et al., 1989; Meik et al., 2008; Montgomery et al., 2013; Nikolakis et al., 2022; Schield et al., 2018, 2019). The hunting behaviors and diets of North American pitvipers are relatively well studied owing to advances in techniques for quantifying the hunting behaviors of free‐ranging individuals (reviewed in Clark, 2016). Furthermore, hunting efficiency in pitvipers is especially relevant to fitness because female reproductive success is tightly linked to feeding (Schuett et al., 2011, 2013; Taylor et al., 2005; Taylor & DeNardo, 2005; Waldron et al., 2013).

Most pitvipers are sit‐and‐wait ambush hunters that use chemosensory cues to locate appropriate ambush sites, where they wait for prolonged periods of time in an attempt to strike and envenomate potential prey (reviewed in Clark, 2016; Nowak et al., 2008; Teshera & Clark, 2021). As with many hunting behaviors, this sequence of events involves a series of complex movements and decisions that are influenced by the behaviors of prey and constraints imposed by environmental conditions; hence, most predatory encounters are not successful. Typically, rattlesnakes striking at small mammals successfully envenomate their prey in less than 50% of encounters (Clark, 2016; Whitford et al., 2017, 2019). Thus, even relatively minor differences in hunting performance could impact the relative fitness of individual snakes.

Mojave rattlesnakes (*Crotalus scutulatus*) and prairie rattlesnakes (*Crotalus viridis*) are known to hybridize in southwestern New Mexico (Zancolli et al., 2016). *Crotalus scutulatus* occupies arid lowland desert habitats (Reynolds & Scott, 1982), typical of the southwestern side of the hybrid zone, while *C. viridis* occupies short‐grass prairie habitats (Holycross, 1993), typical of the northeastern side. As adults, both species hunt and consume small mammals, particularly rodents (Garrigues, 1962; Holycross, 1993; Reynolds & Scott, 1982; Rothe‐Groleau & Fawcett, 2022). However, *C. viridis* also incorporates lizards and, to a lesser extent, amphibians and birds into its diet (Chiszar et al., 1993; Hayes, 1992; Ludlow, 1981; Reed & Douglas, 2002; Stabler, 1948). Both species rely on ambush hunting as their primary strategy for prey capture (Cardwell, 2008; Hayes & Duvall, 1991). Additionally, an experimental study indicated that *C. viridis* exhibits an ontogenetic shift in their preference, favoring lizard prey as juveniles and mammalian prey as adults (Saviola et al., 2012).

To explore how the expression of these complex predatory behaviors may be impacted by hybridization between the two lineages, we integrated a number of approaches to examine the feeding ecology of parental and hybrid individuals. We hypothesized that hybrids would show transgressive patterns of hunting behavior when compared to the parents, with hybrids exhibiting poorer body condition and fewer or less successful prey encounters or lower levels of effort. We also evaluated the hypothesis that hybrids exhibit a transgressive diet, specializing on prey that is either not present in habitats occupied by parental individuals or prey that is typically rejected by parentals. We collected individuals throughout the zone of hybridization and from areas peripheral to the hybrid zone, obtained sex, size, and mass to create an index of body condition, and used genetic approaches to determine individuals' hybrid index. To characterize the hunting behavior of snakes in situ, we tracked individuals via radiotelemetry and monitored hunting behavior using fixed‐field videography. To compare the availability of prey species across different habitats used by snakes, we quantified the relative abundance of small mammals using live trap grids. To examine the diets of individuals, we quantified the relative frequency of mammal and lizard remains in the fecal and stomach contents.

## 2 METHODS

### 2.1 Study sites

Three main study sites were established to study hybrid and parental rattlesnakes. The hybrid (*C. scutulatus* × *viridis*) zone is located within the Cochise Filter Barrier (CFB), a transitional region between the Chihuahuan and Sonoran deserts frequently implicated in lineage divergence due to climatic and vegetation community shifts induced by glacial cycling (Van Devender et al., 1984). Since the CFB is considered a region of “soft” allopatric divergence, gene flow across the barrier is still possible through the dispersal of some individuals across the region (Castoe et al., 2007; Pyron & Burbrink, 2010). Because there is not a major physical barrier separating the two deserts, the CFB has frequent secondary contact between lineages and hybridization between them.

**FIGURE 1:**
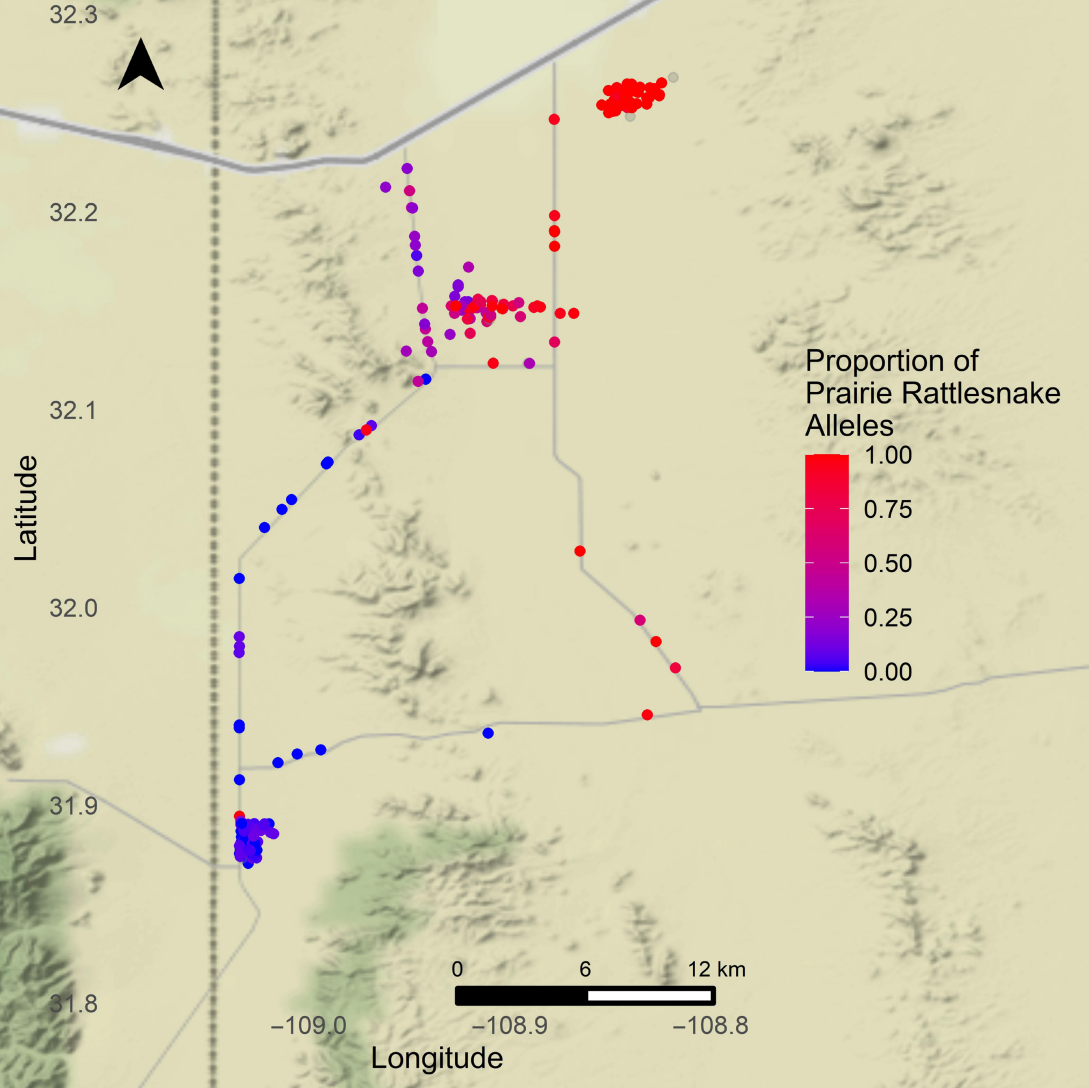
Genetic group and capture location for all genetically sampled snakes (including juveniles, *n* = 189). We classified any snake with a hybrid index (HI) between 5% and 95% as a hybrid. Colored points indicate HI classification (*n* = 189; *Crotalus scutulatus* = 41; *C. viridis* = 60; *C. scutulatus* × *viridis* = 88). Grayed‐out points (*n* = 6) indicate individuals in the study with no HI estimate due to sample failure (Putative *C. scutulatus* = 1; Putative *C. viridis* = 3; Putative *C. scutulatus* × *viridis* = 2).

Within the CFB, the hybrid zone occupies a valley between the Peloncillo and Animas mountains in southwest of New Mexico, U.S.A. (Figure 1). The area contains sporadic homesteads with various amounts of active pasture/agricultural land. Hybrid snakes are found in a narrow band of transitional/mosaic habitat in the center of the valley, with parental populations located on either side of the bordering mountain ranges (Zancolli et al., 2016). The Mojave rattlesnake (*C. scutulatus*) site (31.891703°N, 109.034757°W) was southwest of the hybrid zone and is characterized as a lowland scrub desert macrohabitat. The prairie rattlesnake site (*C. viridis*; 32.259056°N, 108.844943°W) was northeast of the hybrid zone and is dominated by short grass prairie habitat with similar plant species to *C. scutulatus* habitat, except that Mesquite (*Prosopis glandulosa*) is less common and is restricted to a riparian corridor bisecting the site. Within the hybrid zone (32.152532°N, 108.914127°W), in the middle of the valley, the macrohabitat transitions from a Creosote (*Larrea tridentata*)‐dominated lowland desert to an arid short‐grass prairie, similar to the prairie rattlesnake habitat, across a southwest/northeast gradient. Across all three active seasons of data collection, 2019–2021, the average temperature was 28.0°C, ranged from 4.67 to 48.5°C, and had an average total accumulated rainfall between 12.0 and 20.5 cm (https://www.wunderground.com/, station PF01). For a more detailed description of the study sites, see Maag (2023).

### 2.2 Snake sampling and surgical procedures

We collected and sampled all rattlesnakes encountered via surveys within and adjacent to the hybrid zone. Upon capture, we recorded GPS coordinates (precision: ±5 m) and assigned a putative species or hybrid status (SCVI = hybrid, CRSC = *C. scutulatus*, and CRVI = *C. viridis*) to each individual based on species‐typical physical features (e.g., tail banding pattern, head scalation, and facial coloration). These putative designations were later verified/quantified using genetic approaches. At the end of each night, snakes were transported back to a field station in Rodeo, NM. After processing, we released each snake at its exact capture site. Each snake was anesthetized (isoflurane) for processing. While anesthetized, individuals were measured to the nearest mm in snout–vent length (SVL) and body mass to the nearest 0.1 g. Additionally, all snakes were permanently marked with a passive integrated transponder (PIT) tag, sampled for tissue and venom, and measured and photographed for additional data on morphometrics, coloration, and scalation.

A subset of captured snakes was implanted with very high frequency (VHF) radio transmitters (Wildlife Materials SOPI‐2380) so they could be radio‐tracked in situ for the collection of hunting behavior data. While these snakes were anesthetized, we followed a standard surgical procedure (Reinert & Cundall, 1982) to implant miniature VHF radio transmitters into their body cavities. Radio transmitters weighed <5% of the snake's body mass. We released snakes at their point of capture after a 24–72 h recovery period. During recovery, snakes were housed in their own individual containers at a temperature range of 22–26°C and provided water ad libitum. All procedures were approved by the San Diego State University Institutional Animal Care and Use Committee (22‐07‐008C). Animals were collected via a New Mexico Department of Game and Fish Scientific Collection permit (authorization number 3605).

### 2.3 Genetic assignments of individuals to parental species or hybrids

To assign individuals to parental species versus hybrids and quantify the hybrid index of individuals, we analyzed reduced‐representation genomic data. For this, DNA was extracted using standard phenol‐chloroform‐isoamyl methods from tissue samples stored in DNA lysis buffer or snap‐frozen in liquid nitrogen and stored at −80°C. A set of 122 samples was genotyped using a double‐digest RADseq (ddRADseq) approach, which was prepared and sequenced by the University of Minnesota Genome Core. For ddRADseq, the restriction enzymes PstI and MspI were used and sequenced on a total of two NextSeq P2 1 × 100 bp runs. In addition to ddRADseq samples, an additional set of 83 samples was prepared for whole‐genome shotgun sequencing using the Illumina Nextera Flex Library Prep kit and sequenced using an Illumina NovaSeq 6000 (with 150 bp paired‐end reads), targeting a per‐sample coverage of ~20×. We used Trimmomatic v0.39 (Bolger et al., 2014) to quality trim raw read files, and bases with a quality score lower than 20 at either the 5′ or 3′ end were removed. Reads with a read length less than 32 or with quality scores less than 30 were also discarded. We mapped all sequenced samples (both ddRADseq and Whole Genome) to the *C. viridis* reference genome (Schield et al., 2019) with BWA 0.7.17 “mem” (Li & Durbin, 2009) using default settings.

To generate sequence variant files (VCFs) across individuals, we used GATK v4.1.9.0 with the best practices workflow (McKenna et al., 2010). Individual VCF files were generated using “HaplotypeCaller.” To combine individual VCFs, we used the “GenomicsDBimport” tool, followed by “GenotypeGVCFs” to call population variants. Variants were filtered with the “VariantFiltration” tool, keeping only high‐quality, non‐singleton biallelic variants located on reference genome scaffolds assigned to chromosomes. We further excluded variant sites with any of the following characteristics: overlap with annotated repeat elements, map to the Z chromosome and were heterozygous in females, with a minor allele frequency of 0.05 or lower, sites with very high coverage (coverage above the 97.5th quantile) consistent with a copy number variant, or sites with >20% missing data across samples using VCFtools v0.1.17 (Danecek et al., 2011). This filtering approach filters all data (including whole genome data) down to the point where all samples overlap at high frequency, thereby effectively filtering all samples down to genomic regions with high coverage in the ddRADseq samples. Using this dataset, we ran ADMIXTURE v1.3.0 (Alexander et al., 2009) for *K* values ranging from 1 to 10, with 10 iterations per *K* value. We used the *K* = 2 model to infer ancestry coefficients for each individual, which we use as a proxy for hybrid index (HI) scores for each individual.

### 2.4 Scaled mass index

Using the mass and SVL data from adult male and non‐pregnant female snakes, we calculated the body condition of adult snakes using the scaled mass index (SMI), as this has been shown to be a more precise indicator of body condition when comparing individuals across different body sizes (Peig & Green, 2009). We treated each “genetic group” (i.e., *C. scutulatus*, *C. viridis*, and hybrids) as a separate sample when calculating the SMIs. To analyze the relationship between body condition and genetic group, we used a linear model (LM), after verifying normality, with SMI as the dependent variable, and genetic group (determined by its hybrid index) and sex as independent variables. We used these same procedures within the hybrid group (except for replacing the genetic group with the individual HI indices) to examine potential relationships between an individual's HI, and its body condition and sex. We also generated scatterplots of HI and SMI to determine if any non‐linear relationships between these variables were present within the hybrid group.

### 2.5 Fixed‐field videography

**FIGURE A1:**
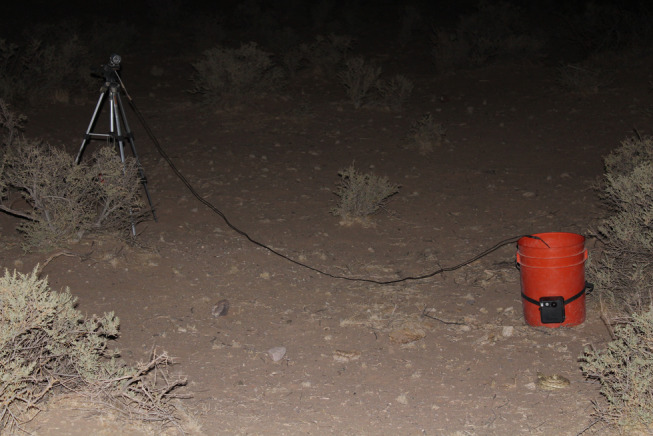
A typical example of the fixed‐field videography unit used to study foraging ecology. The set‐up consists of a near‐infrared (IR)‐sensitive surveillance camera mounted 1 m from a coiled snake, approximately 45° to the left or right side in front of the snake's head, followed by a separate IR light positioned around 3 m away from the front of the snake to illuminate a 1 m^2^ area around the snake. This unit is recording a prairie rattlesnake (*Crotalus viridis*) with the snake in the lower right corner of the photograph.

To gather data on the hunting behavior and diet of individuals, we used a modified version of the fixed‐field videography approach described in Clark (2016). We located free‐ranging individuals implanted with transmitters daily and then deployed videography units to record the behaviors of individuals found in stereotypical ambush coils (Reinert et al., 2011). Field videography units consisted of a near‐infrared (IR)‐sensitive surveillance camera mounted 1 m from a coiled snake, approximately 45° to the left or right side of the head of the snake, depending on the local habitat structure. A separate near‐IR light was positioned ~3 m from the snake to illuminate a 1 m^2^ area with IR light that was visible to the camera but could not be detected by animals (Figure A1). Cameras recorded continuously at 0.5 frames per second (fps) and increased to 1 fps when motion was detected in the frame. Videos recorded in this fashion allowed us to calculate the rates and outcomes of predatory encounters as well as the abandonment times of individuals (snakes in this habitat retreat to thermal refugia during the heat of the day). Cameras were relocated as necessary when snakes changed ambush locations. Video footage was scored independently by two observers blind to the hybrid index of the snakes in order to reduce human error in quantifying relevant metrics. Reviewers quantified chemosensory probing and mouth gaping, abandonment times, prey encounter rates, and outcomes of prey encounters (Clark et al., 2016). Chemosensory probing (extension of the head outside the ambush coil while continuously tongue flicking) is thought to be a mechanism rattlesnakes use to continually reevaluate hunting locations by sampling local chemical cues via the vomeronasal organ (Barbour & Clark, 2012). Mouth gaping (visually similar to yawning) appears to be functionally related to chemosensory probing, apparently serving to clear the vomeronasal organ, located on the roof of the mouth (Graves & Duvall, 1985). Although both behaviors are related to chemosensory behavior and were found to be correlated to one another (Spearman Correlation: *r* = .73, *n* = 40, *p* < .001), we analyzed them independently because they are thought to serve different functions and could potentially be differentially impacted by hybridization. Reviewer scores were averaged to obtain final values; however, when reviewer scores differed in the number or occurrence of snake hunting behaviors, outcomes of prey encounters, or abandonment times, a third individual independently reviewed the video footage. If a mistake was found, then the third reviewer's score was used; otherwise, we averaged the behavioral scores of the third reviewer and the reviewer who had the next closest score to create a final score.

To analyze the relationship between hunting behavior and genetic group, we used LM when the data could be transformed to conform to a normal distribution and showed no signs of having differences in variances across the genetic linages. When either of these assumptions could not be met, we used a generalized linear model (GLM) framework. The individual's genetic group (determined by its hybrid index; see below) was used as the independent variable for all models. For each dependent variable (hunting frequency [proportion of nights that the snake was hunting or hunted while tracked], probing rate, gaping rate, prey encounter rate, strike frequency, successful strike frequency, and abandonment time), we created three models with the following fixed factors: genetic group, genetic group + SMI, and genetic group × SMI. We used AIC_C_ to select which of the three models best fit the data. We included SMI to account for differences in hunting behaviors based on the body condition of the snakes (e.g., a snake with a lower body condition might hunt for longer or more often to try to improve its SMI). When more than one model was within 2 ΔAIC_C_ of the top model, we chose only to analyze the simplest model (the model with the fewest number of independent variables). Last, we used either a Bartlett's test, if the data conformed to normality or could be transformed to normality, or a Levene's test (if the data had a non‐Gaussian distribution), to assess whether the variation between the three groups was equal.

Due to the limited sample of individuals, we did not incorporate sex as a factor in the analysis. Past studies of crotaline hunting behavior indicate that the sexes do not differ in the metrics we calculated (reviewed in Clark, 2016). Additionally, hunting behavior in all three groups was sampled for at least two of the 3 years (effort across groups was haphazard) over which the study took place, with each annual sampling period encompassing the summer active season (May through late August or early September). Year‐to‐year variation in temperature and precipitation patterns for this region was not strong, and the spatial ecology of individuals was generally consistent across years (Maag et al., 2023), leading us to believe that annual variability in environmental conditions would not strongly bias the patterns of hunting behavior we collected across the groups. We calculated hunting effort by counting the proportion of nights that a snake was found on the surface in a stereotypical ambush coil and/or eating a food item. Rates of chemosensory probing, mouth gaping, and prey encounters were calculated from the total amount of video recorded via field videography units for each individual snake. Because past studies on rattlesnakes indicate that probing and gaping rates differ between daytime and nighttime hours (Barbour & Clark, 2012), we conducted separate daytime and nighttime analyses for the rates of these behaviors. A prey encounter was counted when a prey item was seen in the field of view of the camera and was in front of the snake (i.e., in the 180° semicircle around the head of the snake with the head positioned at the midpoint of the semicircle). We calculated the individual rate of predatory strikes as the number of strikes toward a prey item divided by the total number of prey items encountered by that snake, and the successful strike rate as the number of predatory strikes where the snake contacted the prey item divided by the total number of strikes. We calculated the abandonment time of day as the time (to the nearest minute) that the snake left the ambush position and moved out of the frame of the camera. Because most behavioral count data were left skewed and zero‐inflated, we followed the recommendation of Smithson and Verkuilen (2006) and transformed the data using a beta distribution.

### 2.6 Prey availability

To determine if all snakes encountered the same types of prey, we only used the prey encounter frequencies from known prey items. We then grouped together all known prey types encountered by snakes while hunting into the following five categories: (1) non‐predatory birds; (2) kangaroo rats; (3) all other rodents; (4) lizards; and (5) toads. Due to the nature of the data, we created four GLMs using the beta distribution with a zero‐inflation transformation (Smithson & Verkuilen, 2006). Each model had prey encounter frequency as the dependent variable and the following fixed factors: prey + prey: group, prey + prey: group + SMI, and prey + prey: group + SMI + SMI: group. We used AIC_C_ to select which of the three models best fit the data. When more than one model was within 2 ΔAIC_C_ of the top model, we chose only to analyze the simplest model (the model with the fewest number of independent variables).

We used trapping surveys to characterize the abundance of small mammals, which are the most important class of available prey (both parental species are considered small mammal specialists as adults; Holycross, 1993; Ludlow, 1981; Reynolds & Scott, 1982; Salazar & Lieb, 2003; Zancolli et al., 2019). Trap lines (HB Sherman Live Traps 3310A) were deployed for 4–10 consecutive nights across all three of the sites where snakes were monitored with radiotelemetry. Trap lines contained 15–25 trapping stations 15 m apart from each other, each with two traps per station. Traps were opened between 18:30 and 22:00 and closed between 00:00 and 3:40, depending on the time of sunset. Most traps were baited with sterilized sunflower seeds. However, the traps at every fifth station were baited with dry cat food in an attempt to sample carnivorous small mammals (e.g., *Onychomys* spp.). Each small mammal captured was identified to at least the genus level, marked with unique ear tags, and measured for mass, body length, hindfoot length, and tail length.

We calculated an index of small mammal abundance for each trap night and line (number of unique captures/hours of trapping) for each collection site. The data could not be transformed to conform to normality, so we created three GLMs with the index of abundance as the dependent variable and the following combinations of independent variables: site (Mojave site, Prairie site, or hybrid zone), site + prey (kangaroo rat or not), and site × prey. We used AIC_C_ to select which of the three models best fit the data. When more than one model was within 2 ΔAIC_C_ of the top model, we chose only to analyze the simplest model (the model with the fewest number of independent variables).

We conducted visual encounter surveys for herpetofauna (presence and absence of toad and lizard prey species) at all three sites. These surveys were ad hoc, and the effort was broadly similar across the sites. Thus, even though the sampling effort was equivalent between the three collection sites, we consider these comparisons to be tentative.

### 2.7 Diet analysis

While fixed‐field videography for quantifying feeding ecology works well to eliminate bias due to differential digestion of prey (Glaudas et al., 2017), it can suffer from small sample sizes. Thus, we combined video diet data with data from other sources. Fecal samples were collected from animals being held for processing and frozen. We then soaked, thawed, and dried samples in 70% alcohol and examined them under a dissecting microscope to identify hairs, teeth, scales, and other prey remains (Hamilton et al., 2012; Salazar & Lieb, 2003; Weatherhead et al., 2009). We palpated and identified any stomach contents in individuals during post‐capture processing and recorded any incidental feeding observations seen during field monitoring.

We used BORIS v. 7.4.11 to review videos and quantify behaviors (Friard & Gamba, 2016). We used R (v. 3.6.3, 2021) for statistical analysis, using the following packages: tidyverse (Wickham et al., 2019), Hmisc (Harrell & Dupont, 2021), nortest (Gross & Ligges, 2015), MuMIn (Barton, 2020), emmeans (Lenth, 2021), betareg (Cribari‐Neto & Zeileis, 2010), car (Fox & Weisberg, 2019), and ggplot2 (Wickham, 2016). When necessary, we performed post‐hoc multiple comparison tests using a Tukey adjustment. Values are reported as the mean ± 1 SEM (R Core Team, 2021).

## 3 RESULTS

### 3.1 Genetic assignments of individuals

**FIGURE 2:**
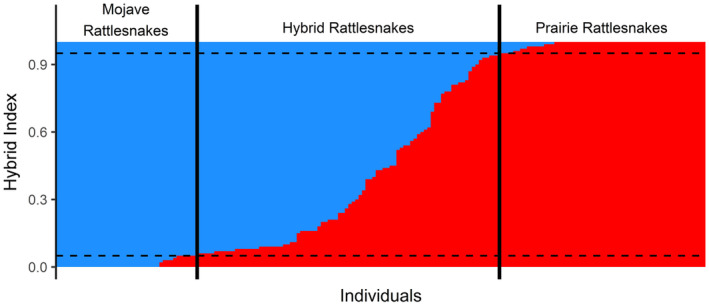
Hybrid index (HI) for all 189 genetically sampled snakes (including juveniles; 41 Mojave, 60 hybrid, and 88 Prairie). Each column is an individual snake, and colors signify the estimated proportion of ancestry from the Mojave rattlesnake (*Crotalus scutulatus*, blue) and prairie rattlesnake (*C. viridis*, red) genomes. The dashed horizontal lines indicate the cutoff used for classifying hybrid individuals (0.05 and 0.95). The solid vertical lines indicate genetic group classifications.

The final genomic VCF dataset, after filtering, contained 189 individuals and 33,071 variant sites. From this dataset, we estimated the ancestry coefficient (using ADMIXTURE) as a proxy of the HI and considered individuals with a HI between 5% and 95% (rounded to the nearest percent) as hybrids (Figures 1 and 2). Based on this, we classified our sampling as including the following numbers of parental and hybrid individuals: *C. scutulatus* = 41, *C. viridis* = 60, and *C. scutulatus* × *viridis* = 88 (Figures 1 and 2).

### 3.2 Scaled mass index

**FIGURE 3:**
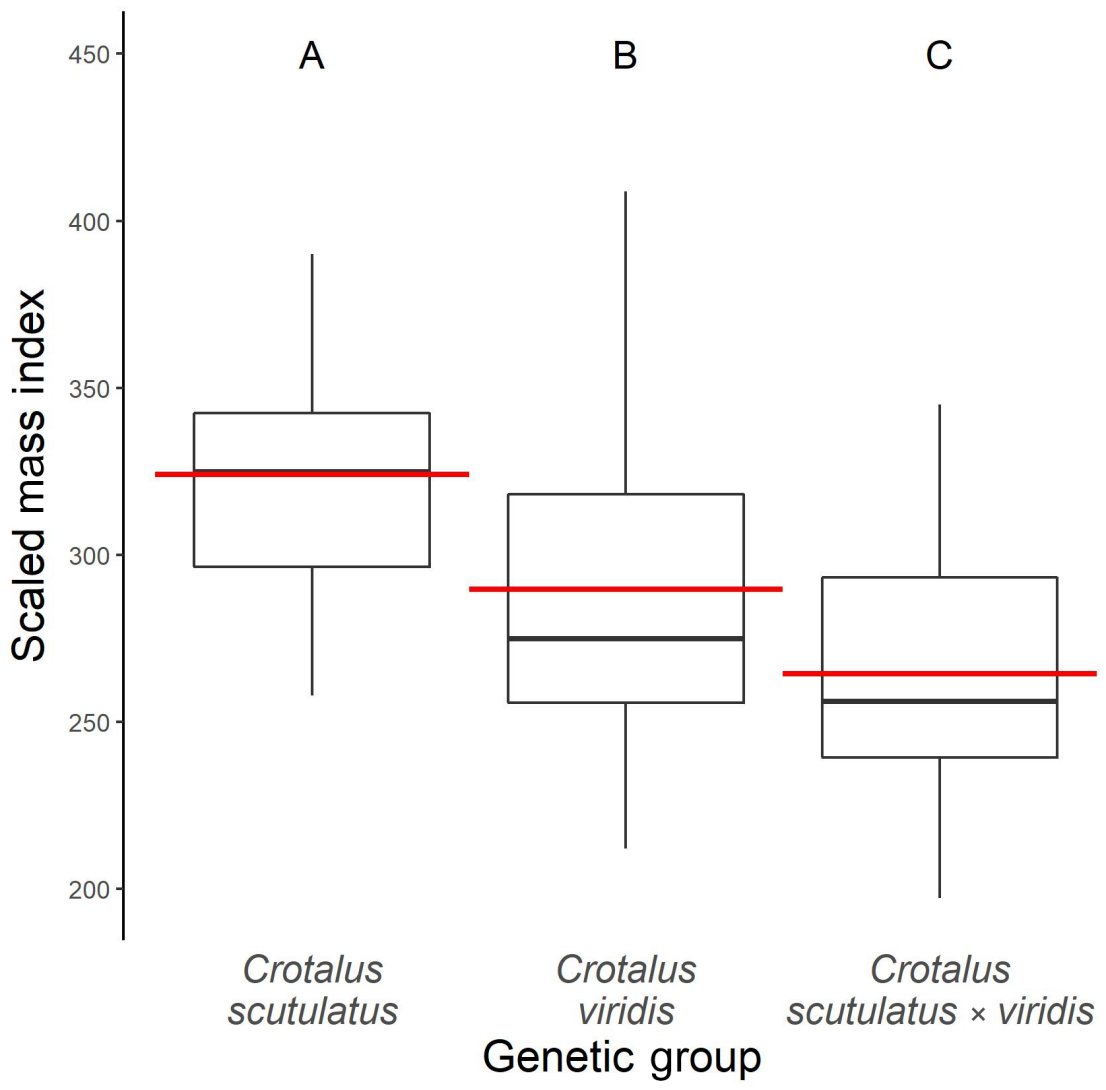
Box plots of body condition (SMI) for adult rattlesnakes. The body condition of snakes in the three genetic groups differed significantly (*F* = 24.1; df = 2,132; *p* < .001). However, no differences in body condition were detected between sexes (*F* = 2.18; df = 1,132; *p* = .142). Mojaves were in better body condition than either prairie or hybrid rattlesnakes (post‐hoc Tukey: *t*‐ratios = 3.76, 6.80; *p* < .001, <.001, respectively), and prairie rattlesnakes were in better condition than hybrids (post‐hoc Tukey: *t*‐ratio = 3.04; *p* = .008). Red lines indicate group means, black lines indicate group medians, the bottom and top of the boxes indicate group first and third quartiles, and the end of the whiskers indicates the largest (top whisker) or smallest (bottom whisker) values within the 1.5 inter‐quartile range from the third and first quartile, respectively. The letters above boxplots indicate statistically significant groupings. Sample sizes: *C. scutulatus* = 36, *C. viridis* = 44, and *C. scutulatus* × *viridis* = 56.

**FIGURE 4:**
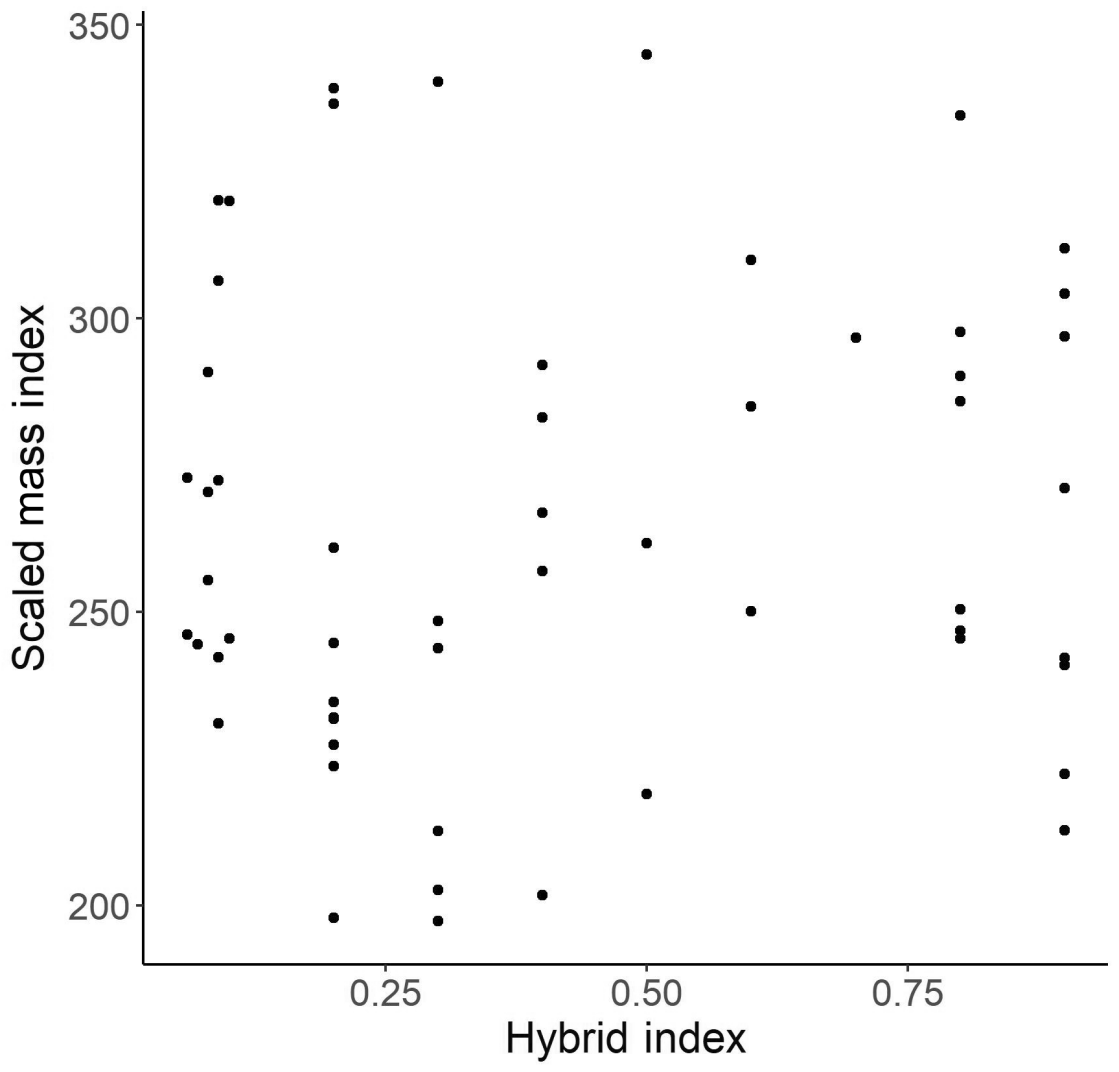
Body condition (SMI) for adult hybrid rattlesnakes in relation to their hybrid index (HI). Body condition of hybrid snakes did not relate to individuals' HI (*F* = 0.670; df = 1,54; *p* = .417). Sample size: 56.

Body condition (SMI) of adult snakes in the three genetic groups (*C. scutulatus*, *C. viridis*, and hybrids) differed significantly (*F* = 24.1; df = 2,132, *p* < .001; Figure 3), but SMI was not different between the sexes (*F* = 2.18; df = 1,132; *p* = .142). Overall, individuals of *C. scutulatus* were in better condition (> SMI) than *C. viridis* or hybrids (post‐hoc Tukey: *t*‐ratios = 3.76, 6.80; *p* < .001, <.001, respectively), and *C. viridis* were in better condition (> SMI) than hybrids (post‐hoc Tukey: *t*‐ratio = 3.04; *p* = .008). Within the hybrid group, the model containing only HI and HI + sex as the predictor variables were the best models to explain the relationship between HI and SMI; therefore, we proceeded with the model containing HI as the sole predictor variable. HI and SMI show no significant relationship to each other within hybrids (*F* = 0.670; df = 1,54; *p* = .417), and visual inspection of the scatterplot between them indicates no non‐linear patterns are present (Figure 4).

### 3.3 Hunting behaviors

Out of the 51 snakes we radio‐tracked, we obtained hunting data through fixed‐field videography on 40 individuals: 16 *C. scutulatus*, 14 *C. viridis*, and 10 *C. scutulatus* × *viridis*. We recorded a mean of 4.8 ± 0.574 hunting nights per snake (2.94 ± 0.403 nights for *C. scutulatus*, 6.07 ± 1.07 nights for *C. viridis*, and 6.00 ± 1.40 nights for *C. scutulatus* × *viridis*).

Snakes in the three genetic groups did not differ in hunting effort. Overall, snakes were found in ambush hunting coils on 60% ± 2.5% of the nights that they were tracked using radiotelemetry. The most informative models analyzing variation in hunting effort were those that contained genetic group and genetic group + SMI, so we report the results of the model with genetic group as the only predictor variable. This model showed that individuals in different groups hunted at an equivalent frequency (*F* = 2.64; df = 2,50; *p* = .081) and also did not differ in the variance of hunting effort (*K* ^2^ = 3.63; df = 2; *p* = .163; Table 1).

**TABLE 1:** Snake hunting behaviors for each group.

Hunting behavior	*Crotalus scutulatus*	*Crotalus viridis*	*Crotalus scutulatus* × *viridis*	Test statistic	*p*‐value
Hunting frequency	0.619 ± 0.045; *n* = 20	0.656 ± 0.031; *n* = 16	0.519 ± 0.047; *n* = 17	*F* = 2.64	.081
Morning probing rate	0.007 ± 0.005; *n* = 10	0.005 ± 0.001; *n* = 13	0.009 ± 0.005; *n* = 9	NA	NA
**Nighttime probing rate**	**0.107 ± 0.009^A^; *n* = 16**	**0.059 ± 0.006** ^**B**^ **; *n* = 14**	**0.080 ± 0.015** ^**AB**^ **; *n* = 10**	***F* = 8.62**	**<.001**
Morning gaping rate	0.003 ± 0.003; *n* = 10	0.001 ± 0.0005; *n* = 13	0.001 ± 0.001; *n* = 9	NA	NA
**Nighttime gaping rate**	**0.020 ± 0.002** ^**A**^ **; *n* = 16**	**0.012 ± 0.002** ^**B**^ **; *n* = 14**	**0.017 ± 0.003** ^**AB**^ **; *n* = 10**	***F* = 3.95**	**.028**
Prey encounter rate	0.004 ± 0.001; *n* = 16	0.005 ± 0.003; *n* = 13	0.003 ± 0.001; *n* = 10	*χ* ^2^ = 0.176	.916
Striking frequency	0.230 ± 0.092; *n* = 13	0.459 ± 0.101; *n* = 11	0.269 ± 0.108; *n* = 9	*χ* ^2^ = 3.36	.187
Successful strike frequency	0.333 ± 0.211; *n* = 6	0.347 ± 0.113; *n* = 10	0.083 ± 0.083; *n* = 6	*χ* ^2^ = 1.32	.518
**Abandonment time**	**05:42 ± 19 min** ^**A**^ **; *n* = 15**	**07:50 ± 37 min** ^**B**^ **; *n* = 14**	**07:16 ± 21 min** ^**AB**^ **; *n* = 10**	***F* = 6.32**	**.004**

*Note*: Hunting Frequency = number of nights a snake was found hunting divided by total nights tracked; probing and gaping rates (probes or gapes/min) = number of probes or gapes divided by total minutes of nighttime or daytime activity; prey encounter rates (prey/min) = number of prey encounters divided by total minutes of hunting activity; strike rate = number of strikes elicited toward a prey item divided by the number of prey encounters; successful strike rate = number of successful strikes (i.e., the recordings show the strike contacting the prey) divided by the number of strikes elicited toward a prey item; abandonment time = the time (to the nearest minute) that the snake left the ambush position and moved out of the frame of the camera. Boldened rows denote significant differences between the groups. Superscripts indicate statistically significant groups by way of post‐hoc multiple comparison tests using a Tukey adjustment.

**FIGURE 5:**
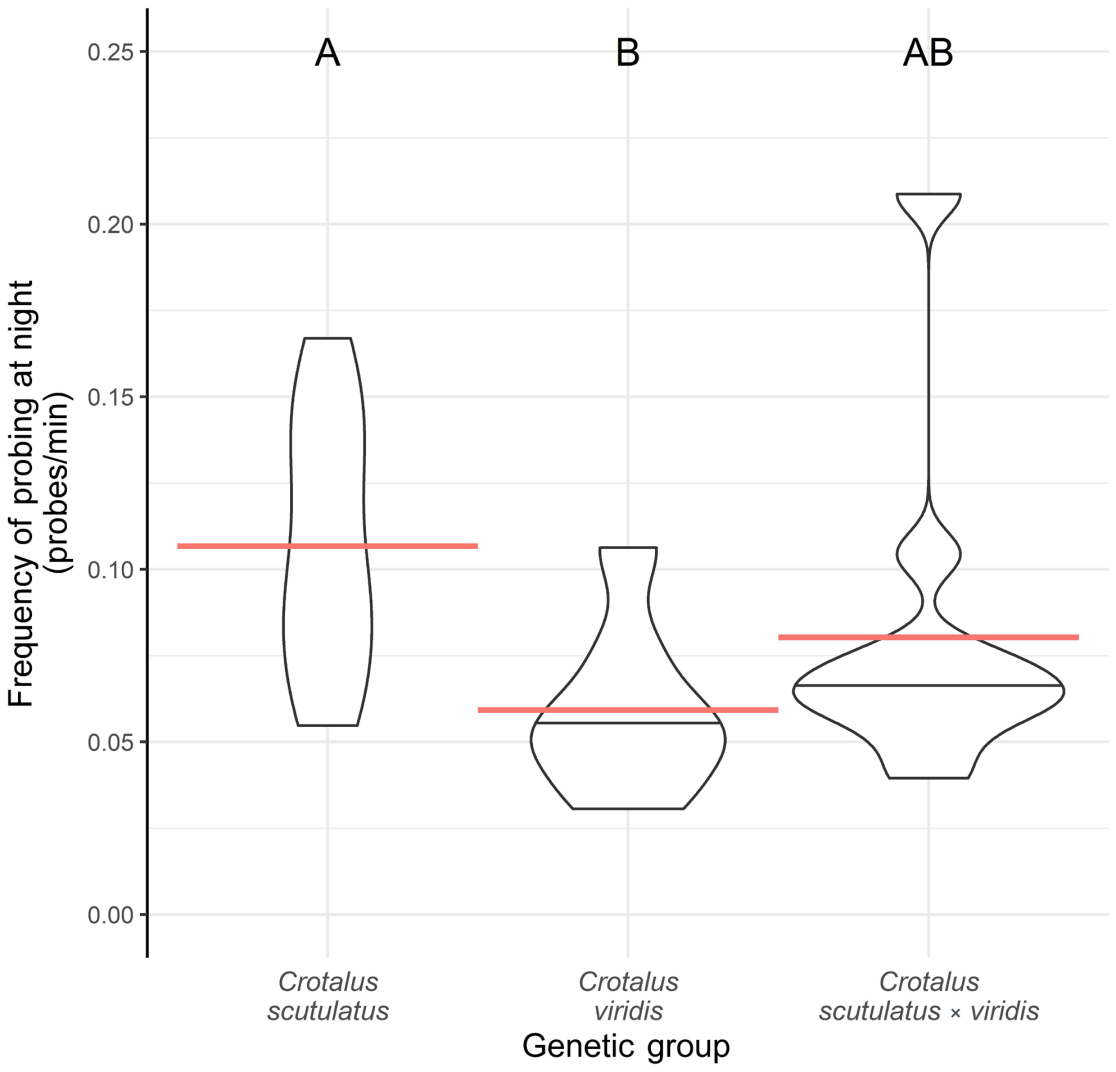
Violin plots of the rate of chemosensory probing while hunting at night in ambush coils. The probing rate for each individual was calculated by dividing the total number of times a snake probed during the nocturnal hours by the total amount of nighttime foraging effort. The genetic groups exhibited different probing rates (*F* = 8.62; df = 2,37; *p* < .001). *Crotalus scutulatus* probed more often than *C. viridis* (post‐hoc Tukey: *t*‐ratio = 4.14, *p* < .001). *C. scutulatus* × *viridis* were no different from either parental group (*C. scutulatus*: *t*‐ratio = 2.09, *p* = .106; *C. viridis*: *t*‐ratio = −1.62, *p* = .249). Variance was not different between the groups (*K* ^2^ = 0.515; df = 2; *p* = .773). Red lines indicate group means. The letters above violin plots indicate statistically significant groupings. Sample sizes: *C. scutulatus* = 16, *C. viridis* = 14, and *C. scutulatus* × *viridis* = 10.

Snakes in different genetic groups did exhibit differences in the frequency of chemosensory probing and mouth gaping while nocturnally hunting. The most informative model for nocturnal probing was the one that contained genetic groups after a log‐transformation of the data. Overall, the three genetic groups exhibited significantly different nighttime probing rates (*F* = 8.62; df = 2,37; *p* < .001). *C. scutulatus* exhibited 0.107 ± 0.009 probes per min or one probe every 9.37 min. This was significantly more frequent than probes of *C. viridis*, which probed at a rate of 0.059 ± 0.006 probes per min or one probe every 16.9 min (post‐hoc Tukey: *t*‐ratio = 4.14, *p* < .001). The probe rate of hybrid snakes (0.080 ± 0.015 probes per min or one probe every 12.5 min) was intermediate and not significantly different from either *C. scutulatus* (post‐hoc Tukey: *t*‐ratio = 2.09, *p* = .106) or *C. viridis* (post‐hoc Tukey: *t*‐ratio = −1.62, *p* = .249; Figure 5; Table 1).

**FIGURE A2:**
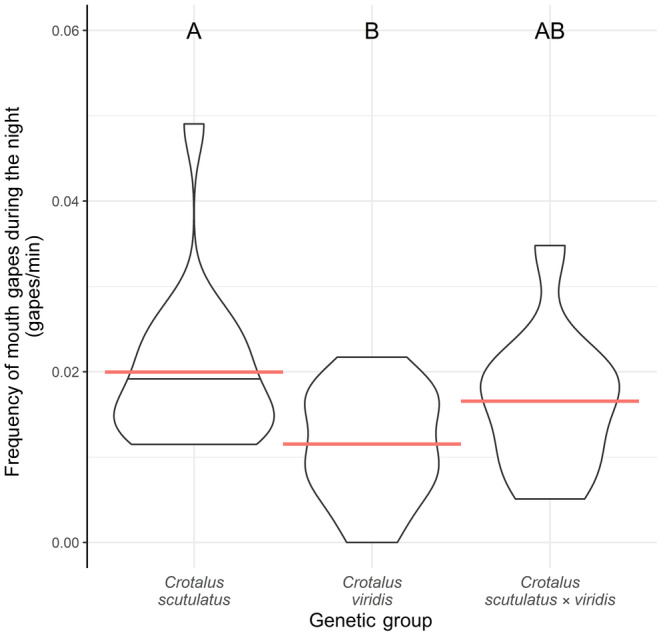
Violin plots of mouth gaping while hunting at night on the surface of the habitat. The gaping rate for each individual was calculated by dividing the total number of times a snake mouth‐gaped during the nocturnal hours by the total amount of nighttime foraging effort. The genetic groups displayed different gaping rates (*F* = 3.95; df = 2,37; *p* = .028). *Crotalus scutulatus* gaped more often than *C. viridis* (post‐hoc Tukey: *t*‐ratio = 2.80; *p* = .021) but not *C. scutulatus* × *viridis* (post‐hoc Tukey: *t*‐ratio = 1.03, *p* = .565), which were no different from each other (post‐hoc Tukey: *t*‐ratio = −1.48, *p* = .313). Variance was not different between the groups (*K* ^2^ = 1.80; df = 2; *p* = .406). Red lines indicate group means. The letters above violin plots indicate statistically significant groupings. Sample sizes: *C. scutulatus* = 16, *C. viridis* = 14, and *C. scutulatus* × *viridis* = 10.

In the analysis of nocturnal mouth gaping rate, the most informative models contained genetic group and genetic group + SMI. Therefore, we report the results of the model containing only genetic group as a predictor variable. The rate of mouth gaping differed between the three genetic groups (*F* = 3.95; df = 2,37; *p* = .028; Figure A2). We found that *C. scutulatus* gaped around 1.74 times more often (0.020 ± 0.002 gapes per min or one gape every 50 min) than *C. viridis* (post‐hoc Tukey: *t*‐ratios = 2.80; *p* = .021), which gaped 0.012 ± 0.002 times per min (once every 87 min). Hybrid snakes gaped 0.017 ± 0.003 times per min or once every 60 min and were not statistically different than the other two groups (post‐hoc Tukey: *t*‐ratios = 1.03, −1.48; *p* = .565, .313; respectively). We found no difference in the variance of both nocturnal probing and gaping rates between the groups (*K* ^2^ = 0.515, 1.80; df = 2; *p* = .773, .406; respectively). Daytime probing and mouth gaping rates for all snakes were extremely low compared to nighttime rates (0.007 ± 0.002 per minute or one probe per 2.5 h; 0.002 ± 0.001 per min or one gape per 11 h; Table 1), and small samples precluded statistical comparisons for daytime rates.

The three genetic groups did not differ in prey encounter rates or outcomes. For prey encounter and strike rates, the most informative model set included the simplest model (genetic group as the sole predictor variable). Strike success rates were best explained by models containing genetic group or genetic group + SMI. For all three metrics, we report the results from models with genetic group as their sole predictor variable. The three groups were not statistically different in prey encounter rates (*χ* ^2^ = 0.176; df = 2; *p* = .916), strike rates (*χ* ^2^ = 3.36; df = 2; *p* = .187), or successful strike rates (*χ* ^2^ = 1.32; df = 2; *p* = .518). We also found no differences in the variances of these hunting metrics between genetic groups (Prey encounter rate: *F* = 0.403; df = 2,36; *p* = .671; Strike rate: *F* = 0.038; df = 2,30; *p* = .963; Successful strike rate: *F* = 1.31; df = 2,19; *p* = .294; Table 1). Overall, snakes encountered 0.004 ± 0.001 prey per min, or one prey item every 4.29 h while hunting. During these prey encounters, snakes struck 31.7% ± 5.87% of the time. Snake strikes were successful (i.e., the strike contacted the prey item) 27.1% ± 8.03% of the time. Hence, for every hour of hunting effort, there is a ~2% probability that the snake will successfully strike a prey item.

**FIGURE 6:**
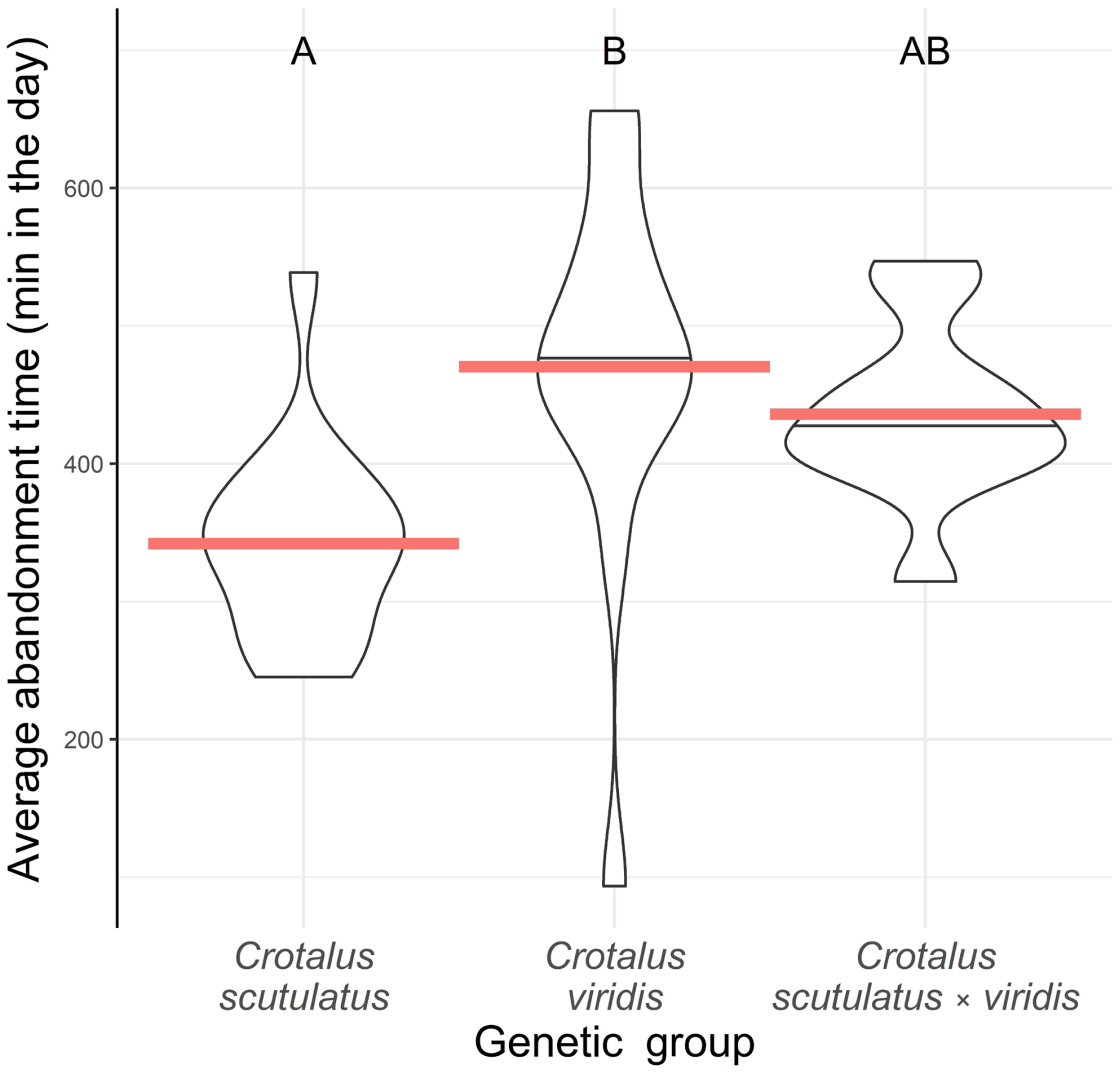
Violin plots of the average time of day (minutes after midnight) that individual snakes abandoned their ambush coils. The genetic groups differed in abandonment time (*F* = 6.32; df = 2,36; *p* = .004). *Crotalus scutulatus* abandoned ambush sites at an average of 5:42, earlier than *C. viridis* (post‐hoc Tukey: *t*‐ratio = −3.45, *p* = .004) but not *C. scutulatus* × *viridis* (post‐hoc Tukey: −2.30, *p* = .069). *C. viridis* and *C. scutulatus* × *viridis* were no different from each other (post‐hoc Tukey: 0.829, *p* = .688), abandoning on average at 7:50 or 7:16, respectively. Variance was different between the groups (*K* ^2^ = 7.60; 2,36; *p* = .022), with *C. viridis* exhibiting the highest level of variance. However, post‐hoc multiple comparisons were inconclusive in differentiating the variance values of the genetic groups after a Holm's adjustment (Holm's adjusted *p* = .482, .383, .072; *C. scutulatus* vs. *C. viridis*; *C. viridis* vs. hybrids; and *C. scutulatus* vs. hybrids, respectively). Red lines indicate group means. The letters above violin plots indicate statistically significant groupings. Sample sizes: *C. scutulatus* = 15, *C. viridis* = 14, and *C. scutulatus* × *viridis* = 10.

Individuals of different genetic groups abandoned their hunting sites (ambush coils) at different times during the morning. Because the most informative models for ambush coil abandonment times contained the genetic group and genetic group + SMI of the snakes as the predictor variables, we reported the results of the model containing genetic group as the only predictor variable. The three groups differed in the time of day they abandoned ambush sites to seek thermal refuge (*F* = 6.32; df = 2,36; *p* = .004; Figure 6). We found that *C. scutulatus* abandoned hunting locations earliest (average at 05:42), but were only significantly different from *C. viridis* (post‐hoc Tukey: *t*‐ratio = −3.45 *p* = .004). *C. viridis* and hybrids left ambush sites a couple of hours later (average time of abandonment at 07:50 and 07:16, respectively). However, hybrids were not statistically different in their abandonment time than either *C. scutulatus* or *C. viridis* (post‐hoc Tukey: *t*‐ratio = −2.30, 0.829; *p* = .069, .688, respectively). Variances of abandonment times between the groups were different (*K* ^2^ = 7.60; 2,36; *p* = .022), but we were not able to differentiate the genetic groups after post‐hoc multiple comparisons and a Holm's adjustment (Holm's adjusted *p* = .482, .383, .072; *C. scutulatus* vs. *C. viridis*; *C. viridis* vs. hybrids; and *C. scutulatus* vs. hybrids, respectively).

### 3.4 Prey availability

**FIGURE A3:**
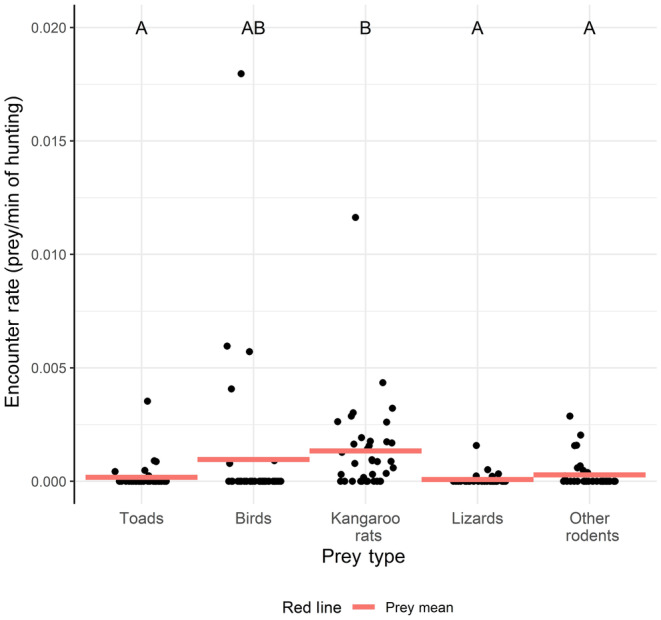
Strip chart of prey encounter rates. Overall, rattlesnakes in the three groups encountered prey types at different rates (*χ* ^2^ = 20.1; df = 4; *p* < .001). Kangaroo Rats (*Dipodomys* spp.) were encountered significantly more than all other prey types (toads, lizards, and other rodents; post‐hoc Tukey: *z*‐ratios = −3.55, 3.83, 3.15; *p* = .004, .001, .014; respectively), except for birds (post‐hoc Tukey: *z*‐ratio = −2.23, *p* = .168), while all other prey items were encountered by rattlesnakes similarly (post‐hoc Tukey: *z*‐ratios = −1.32, 0.286, −0.397, 1.6062, 0.924, −0.683; *p* = .678, .999, .995, .493, .888, .960). Red lines indicate the mean encounter rates of the given prey type for 37 rattlesnakes. The letters above box plots indicate statistically significant groupings.

The most informative models for prey encounters contained only prey type as the predictor variable, indicating that genetic groups did not differ in the type of prey encountered while hunting. As a whole, snakes encountered different types of prey (kangaroo rats, lizards, toads, birds, and other rodents) at different rates (*χ* ^2^ = 20.1, df = 4, *p* < .001, Figure A3). Kangaroo rats were encountered significantly more often than all other prey types (toads, lizards, and other rodents; post‐hoc Tukey: *z*‐ratios = −3.55, 3.83, 3.15; *p* = .004, .001, .014, respectively), except for birds (post‐hoc Tukey: *z*‐ratio = −2.23, *p* = .168). All other prey were encountered similarly (post‐hoc Tukey: *z*‐ratios < 1.61; *p* > .493). Even though birds were frequently recorded with our camera traps, snakes were never observed striking at them, and they were not present in their fecal or gut contents (see below). Accordingly, we do not consider birds to be important prey for the three genetic groups studied at these sites, even though other populations have found birds to be a minor component of the diet of *C. viridis* (Hayes, 1992; Ludlow, 1981). All other encountered known prey types were struck by snakes at similar rates. Snakes struck 33.8% of the kangaroo rats encountered, 44.4% of toads encountered, 26.7% of other small mammals encountered, and 12.5% of lizards encountered. Snakes were also equally successful at striking all prey types (~1/3 strikes were successful). Kangaroo rats were successfully struck in eight out of the 24 attempts; toads were successfully struck two out of the four attempts; other small mammals were struck in one out of the four attempts; and one strike against a lizard was not successful.

**FIGURE A4:**
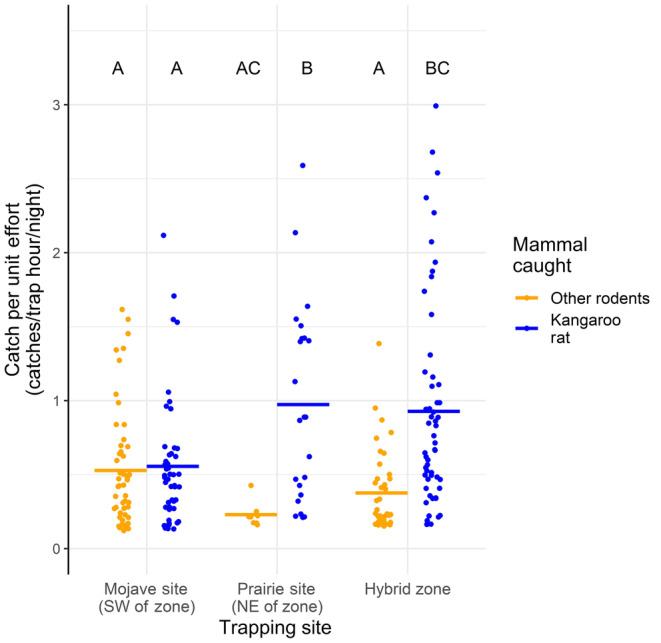
Strip chart of the nightly catch per unit effort for small mammal prey types surveyed at each of the three sites. No difference was found between the sites in overall small mammal abundance (*χ* ^2^ = 4.09; df = 2; *p* = .130). Kangaroo rats (*Dipodomys merriami*, *D. ordii*, and *D. spectabilis*) were captured 1.8 times more often than all other rodent species (*χ* ^2^ = 28.3; df = 1; *p* < .001), but this pattern differed by site (site: prey type, *χ* ^2^ = 20.0; df = 2; *p* < .001). The site SW of the hybrid zone used by Mojave rattlesnakes had an equal abundance of kangaroo rats and other rodents (post‐hoc Tukey: *z*‐ratio = 0.337, *p* = .999), whereas kangaroo rats were 2–4 times more common than all other rodents at other sites (post‐hoc Tukey: *z*‐ratios = 2.87, 4.73; *p* = .047, <.001; respectively). Line segments indicate the mean catch per unit effort for each site across all trapping nights.

The most informative model of small mammal abundance included collection site, prey category (kangaroo rat species or other rodent species), and their interaction as predictor variables. Small mammal trapping yielded similar abundance of rodent species across the three sites (*χ* ^2^ = 4.09; df = 2; *p* = .130). Reflecting the predatory encounter rates, kangaroo rats were captured 1.8 times more often than all other rodent species (*χ* ^2^ = 28.3; df = 1; *p* < .001). We did find a significant interaction between the relative abundance of kangaroo rats and the trapping site (*χ* ^2^ = 20.0; df = 2; *p* < .001; Figure A4). The Mojave rattlesnake site had an equal abundance of kangaroo rats and all other rodent species combined (post‐hoc Tukey: *z*‐ratio = 0.337, *p* = .999), while the other two sites exhibited 2–4 times more kangaroo rats than all other rodent species combined (post‐hoc Tukey: *z*‐ratios = 2.87, 4.73; *p* = .047, <.001; respectively).

Visual encounter surveys for small lizards and toads that represent prey items revealed no major differences between the three sites (Table 2). The lizard and toad species richness between the sites are almost even, with 10 species present at each of the study sites for *C. scutulatus* and *C. viridis*, and 12 at the hybrid site. Although we were not able to estimate the abundance of each species, qualitatively, we did not see major differences in lizard or toad abundance.

**TABLE 2:** Presence/absence data of toad and lizard species at field sites southwest of the hybrid zone (Mojave site), northeast of the hybrid zone (Prairie site), and within the hybrid zone.

Site	Year	Toads						Lizards							
		Great Plains Toad (*Anaxyrus cognatus*)	Green Toad (*A. debilis*)	Red‐spotted Toad (*A. punctatus*)	Woodhouses Toad (*A. woodhouseii*)	Couch's Space foot (*Scaphiopus couchii*)	Desert Spadefoot (*Spea multiplicate*)	Whiptails (*Aspidoscelis* spp.)	Western Banded Gecko (*Coleonyx variegatus*)	Long‐Nosed Leopard Lizard (*Gambelia wislizenii*)	Elegant Earless Lizard (*Holbrookia elegans*)	Texas Horned Lizard (*Phrynosoma cornutum*)	Round‐tailed Horned Lizard (*P. modestum*)	Fence Lizards (*Sceloporus* spp.)	Side‐Blotched Lizard (*Uta stansburiana*)
Mojave site (southwest of zone)	2021	P	P	P	P	P	P	P	P	A	A	P	A	P	A
Prairie site (northeast of zone)	2020	P	P	A	A	A	P	A	P	P	A	P	P	A	A
	2021	A	P	A	P	P	A	P	P	P	A	P	P	A	A
Hybrid zone	2019	P	P	A	A	P	P	P	P	A	P	P	P	P	P
	2020	A	A	A	A	A	A	A	P	P	A	P	P	A	A
	2021	P	P	A	A	P	A	P	P	A	A	P	P	A	P

*Note*: A, species was not detected; P, species was detected.

### 3.5 Stomach contents and fecal samples

**FIGURE 7:**
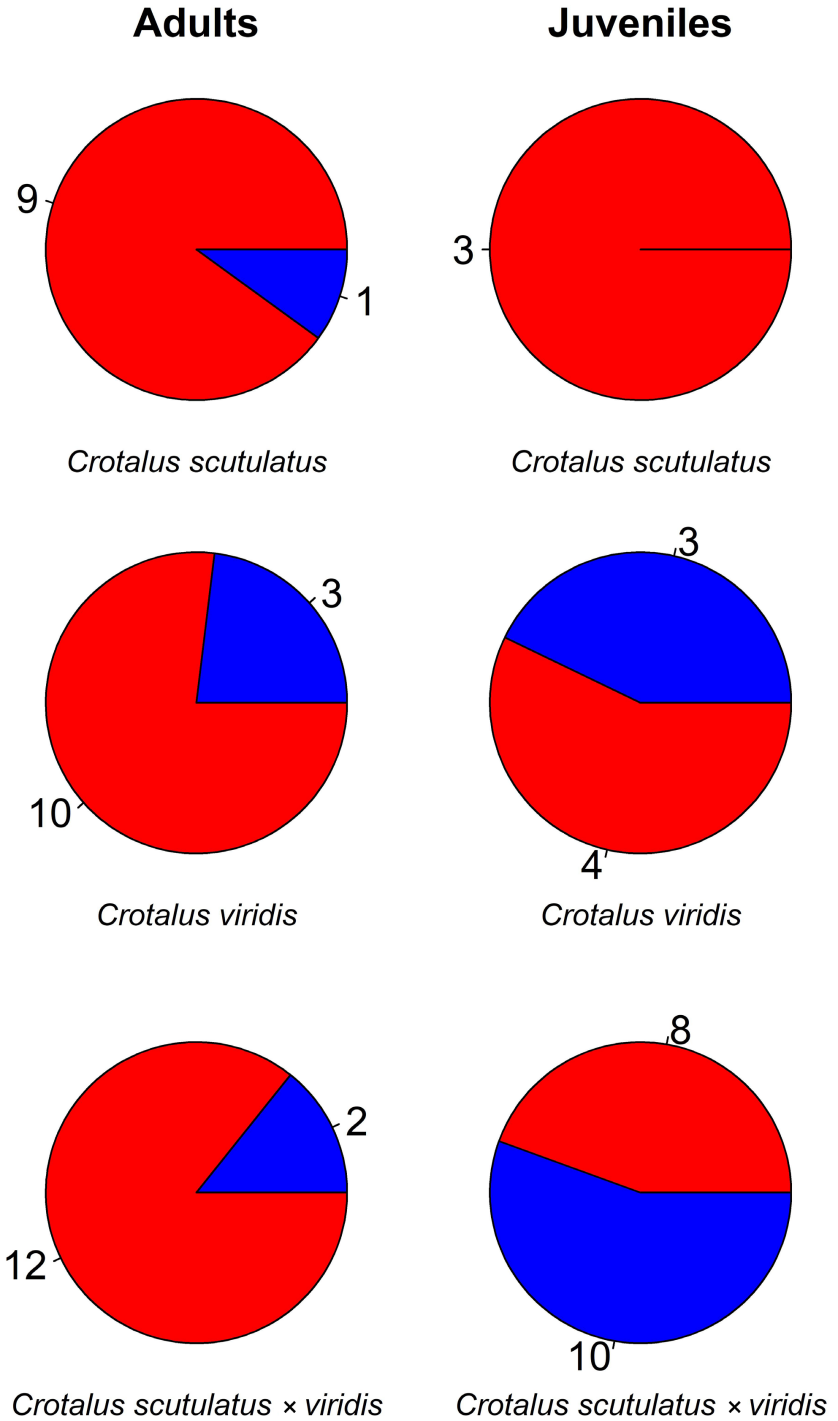
Pie charts of the proportion of mammalian and lizard remains found in the fecal samples and stomachs of 60 parental and hybrid rattlesnakes. Red indicates mammal remains; blue indicates lizard remains. The numbers around pie charts indicate sample sizes for the prey types (lizards or mammals) within groups. Snake sample sizes: Adult *Crotalus scutulatus* = 9, juvenile *C. scutulatus* = 2, adult *C. viridis* = 12, juvenile *C. viridis* = 5, adult *C. scutulatus* × *viridis* = 12, and juvenile *C. scutulatus* × *viridis* = 13.

We collected and analyzed fecal samples from a total of 33 adult rattlesnakes (*C. scutulatus* = 9, *C. viridis* = 12, hybrids = 12) and 20 juveniles (*C. scutulatus* = 2, *C. viridis* = 5, hybrids = 13; Table A1). We palpated seven discrete prey items from the stomachs of anesthetized snakes (Table A2). These samples resulted in a total of 66 individual prey items, and all were mammals (*n* = 46) or lizards (*n* = 20; Figure 7). Due to sample size limitations within each of the three genetic groups, we did not perform any statistical tests between the groups. However, snakes of all groups had similar diets within age classes (Figure 7). When compared as a whole (i.e., all groups combined), adults and juveniles differed in diet (*χ* ^2^ = 5.65; df = 1; *p* = .017). Juveniles fed equally on lizards and mammals as prey (53.6% of prey items of juveniles are small mammals), whereas adults shifted to a diet primarily of small mammals (83.8% of prey items of adults are small mammals).

## 4 DISCUSSION

Overall, the hunting behavior, prey availability, and diet of Mojave rattlesnakes (*C. scutulatus*), prairie rattlesnakes (*C. viridis*), and hybrids were remarkably similar, with only minor differences among them. Snakes at the three study sites exhibited comparable rates of hunting behaviors, encountered and successfully subjugated prey at similar rates, and had broadly overlapping diets. However, we found that the body condition index of hybrids was significantly lower than that of individuals of either parental species. This pattern indicates that factors other than differences in hunting behavior or diet may underlie the relatively poorer body condition of hybrid snakes.

The rate of prey ingested by rattlesnakes, particularly females, can drastically affect their reproductive success (Schuett et al., 2011, 2013; Taylor et al., 2005; Taylor & DeNardo, 2005; Waldron et al., 2013). Thus, differences in the hunting efficiency and diet of individuals can result in differences in their relative fitness. However, counter to our hypothesis, hybrid rattlesnakes did not exhibit transgressive patterns of hunting behavior. Rather, hybrids were generally not different from parental individuals, displaying intermediate values in metrics where the parental groups differed from each other.

For example, hybrid rattlesnakes exhibited intermediate rates of chemosensory behavior while in ambush, with *C. scutulatus* and *C. viridis* differing from each other (rates of chemosensory probing and gaping, Figures 5 and A3). Our study represents the first direct comparison of the rates of these behaviors across populations or species. Surprisingly, we found that *C. scutulatus* had consistently higher frequencies of chemosensory probing and mouth gaping than did *C. viridis*, even though they occupied very similar habitats and relied on similar types of prey. It is unclear why this might be the case, but it may be indicative of some subtle underlying differences in sensory systems that are not yet understood. Although comparative data on interspecific variation in the sensory systems of pitvipers are generally lacking, the external and internal anatomy of their facial pits (IR sensory organs) does vary among taxa. The anatomy of the facial pits affects their sensory fields and causes small differences between species in the spatial resolution of the system (Bakken et al., 2012). Thus, it is possible that *C. viridis* differs from *C. scutulatus* in some aspects of sensory acuity (e.g., visual) that influence their relative frequency of investigation while in ambush. Although increased movements associated with chemosensory probing might be expected to make individuals less cryptic, we found no evidence of a functional tradeoff associated with higher rates of chemosensory behaviors: active *C. scutulatus* exhibited similar encounter rates and outcomes with potential prey (Table 1) and encountered the fewest predators compared to *C. viridis* and hybrids (Maag & Clark, 2022).

Encounters with prey species and the outcomes of those encounters were similar across the three rattlesnake groups (Table 1). Given that all three study sites also had an equivalent abundance of small mammals (Figure A4), hybrid snakes seem to be as effective as parentals at locating ambush sites. Furthermore, hybrid rattlesnakes exhibited similar strike rates and strike success rates when encountering prey (Table 1), although the sample size is insufficient to make robust comparisons. However, it is likely that larger samples of relevant data would require a more experimental context, such as staged predatory encounters in captive or semi‐natural enclosures.

In both parental species and hybrids studied, kangaroo rats were the most frequently encountered prey type (Figure A3). The success rate of rattlesnakes in our study when striking toward kangaroo rats (33.3%) was also similar to strike success rates observed in sidewinder rattlesnakes (*Crotalus cerastes*) attacking kangaroo rats (34.8%–46.9%; Whitford et al., 2017, 2019). The similarities between the primary prey (kangaroo rats) and hunting efficiency for *C. cerastes*, *C. scutulatus*, *C. viridis*, and the hybrids we studied suggest that rattlesnake hunting behaviors and success rates may be relatively conserved across species.

We did find that the three groups differed in their abandonment times, indicating a significant difference in daytime hunting frequencies. Mojave rattlesnakes would abandon their ambush coils earlier in the morning than *C. viridis* (hybrids were not statistically different from either parental group; Figure 6). The small mammals that make up the bulk of the diet of all three groups are largely nocturnal, whereas lizards eaten occasionally by these snakes are exclusively diurnal. Thus, the tendency to remain in ambush into the daylight hours exhibited by *C. viridis* (and, to a lesser extent, hybrids) might reflect an increased reliance on lizards as prey items. The preliminary data from dietary analyses support this pattern (Figure 7), as adult *C. scutulatus* had the lowest proportion of lizards in their diet overall. However, dietary data were relatively limited in sample size, and statistical analyses of these patterns would require a larger sample of fecal or stomach contents. Nevertheless, the initial pattern in both behavior and diet indicates a potentially important difference between the groups, with *C. scutulatus* showing increased reliance on mammalian prey resources and *C. viridis* showing a stronger tendency to use morning hours to hunt diurnal lizards.

We also found that the relative abundance of small mammals (Figure A4) and richness of the ectothermic prey types (toads and lizards, Table 2) were similar between each of the prey communities at the three study sites. At all three sites, kangaroo rats were the most abundant rodents in both trapping surveys and field encounters with snakes. Both of the parental species we studied are frequently characterized as rodent generalists as adults, with an ontogenetic shift away from lizards as prey of juvenile snakes (Garrigues, 1962; Holycross, 1993; Reynolds & Scott, 1982; Rothe‐Groleau & Fawcett, 2022), and our data generally supports this pattern. Further exploration of the potential differences between these two species and their hybrids would require more detailed behavioral data on the foraging ecology of juvenile snakes—data that are generally lacking due to constraints on the size of radio transmitters—as well as an increased sample size for each of these.

Some shortcomings of our approach to quantifying hunting behaviors are inherent to fixed‐field videography. Once snakes struck and released prey items, they generally left the field of view of the camera while using chemosensory trailing behavior to locate and ingest prey carcasses. Thus, the frequency and timing at which prey succumbed to venom are unknown. Prey can survive envenomation through either physiological venom resistance (Robinson et al., 2021) or rapid escape from bites, lowering the time the snake has to inject venom (Whitford et al., 2017, 2019). The process of chemosensory trailing to locate prey carcasses can also be prolonged and occasionally lead to failure on the part of the snake to locate the carcass (Teshera & Clark, 2021). Thus, it is possible that differences exist between the three groups at these stages of the hunting process, and additional field methodologies would be required to evaluate this possibility (such as animal‐borne accelerometry; see Hanscom et al., 2023).

Overall, the snakes from parental lineages hybridizing in this area are generally similar in their hunting behaviors and diet, with hybrids largely resembling parentals (or exhibiting intermediate values) in different metrics of foraging ecology. Comparable patterns have been reported in other hybrid systems, with hybrids exhibiting similar or intermediate hunting or foraging behaviors when compared to parental individuals (Peters & Kleindorfer, 2015; Sas et al., 2005; Vamosi et al., 2000). The general pattern of hybrids using prey resources or exhibiting foraging behaviors that match parental species could have a number of implications for understanding the factors that may influence the dynamics of the hybrid zone. In systems where food resources drive spatial behaviors, hybrids and parentals that exhibit similarities in foraging ecology could encounter each other more frequently, leading to increased back‐crossing of hybrids with one parental species. However, when individuals are found in multiple types of habitats with variable food resources, then habitat type, rather than group per se, would be expected to drive variation in feeding behaviors. For example, hybrid woodrats (*Neotoma bryanti* × *N. lepida*) were found to have diets that were more dependent on habitat than ancestry (Nielsen et al., 2023). Neither of these factors appears to be a major extrinsic barrier to hybridization in the Mojave/prairie rattlesnake hybrid zone we studied, as we found that the prey communities are similar in abundance and composition across the region. Additionally, individuals from all three genetic groups (parental species and hybrids) have been found within our central study site location, implying that hybrid individuals spatially overlap with both parental types. Thus, it is unlikely that hunting behavior or feeding ecology shape hybridization dynamics in this snake system. Our findings that hybrids were in significantly poorer body condition when compared to parental species individuals, despite similar hunting and foraging behaviors to parentals, suggest that other intrinsic metabolic or physiological incompatibilities may exist in these hybrids. Future studies to further investigate the nature of these potentially intrinsic impacts on hybrids could help develop a more general understanding of hybridization dynamics in animals.

## AUTHOR CONTRIBUTIONS

**Dylan W. Maag:** Conceptualization (lead); data curation (lead); formal analysis (lead); funding acquisition (equal); investigation (lead); methodology (lead); project administration (equal); resources (lead); software (lead); supervision (equal); validation (lead); visualization (lead); writing – original draft (lead); writing – review and editing (equal). **Yannick Z. Francioli:** Data curation (supporting); writing – original draft (supporting); writing – review and editing (supporting). **Noelle Shaw:** Data curation (supporting); investigation (supporting); writing – review and editing (supporting). **Ashana Y. Soni:** Data curation (supporting); investigation (supporting); writing – review and editing (supporting). **Todd A. Castoe:** Writing – original draft (supporting); writing – review and editing (supporting). **Gordon W. Schuett:** Conceptualization (supporting); methodology (supporting); project administration (supporting); writing – review and editing (supporting). **Rulon W. Clark:** Conceptualization (supporting); funding acquisition (equal); investigation (supporting); methodology (supporting); project administration (equal); supervision (equal); visualization (supporting); writing – original draft (supporting); writing – review and editing (equal).

## CONFLICT OF INTEREST STATEMENT

The authors declare no conflicts of interest.

## Data Availability

Raw sequence reads for RADseq data are available under the NCBI SRA (sequence read archive) BioProjectID number: PRJNA1010815. The final VCF alignment file used for hybrid index analysis, snake SMI, hunting behavior, hunting frequency, prey encounter, toad and lizard presence/absence, and small mammal trapping data are available on Dryad: https://doi.org/10.5061/dryad.ghx3ffbvc.

## APPENDIX A

**TABLE A1:** Fecal sample data.

Snake ID	Genetic group	HI	Sex	Age	Snake mass	SVL	Date feces collected	Fecal dry mass	Mammal remains	Lizard remains
CRSCA21	SCVI	0.09	F	J	99.9	533	5/21/2021	1.43	Y	N
CRSCAA21	CRSC	0.00	F	A	219.7	679	7/16/2021	6.91	Y	N
CRSCBB21	CRSC	0.00	M	A	667.6	900	7/16/2021	13.1	Y	N
CRSCCC21	SCVI	0.07	M	J	64.4	470	7/30/2021	0.74	Y	Y
CRSCD21	CRSC	0.00	M	A	333	746	6/5/2021	3.08	Y	N
CRSCD21	CRSC	0.00	M	A	333	746	9/13/2021	2.83	Y	N
CRSCDD21	CRSC	0.00	F	A	268.8	692	7/18/2021	7.56	Y	N
CRSCE21	SCVI	0.07	F	J	47.2	397	6/9/2021	1.65	Y	N
CRSCEE21	CRSC	0.05	F	J	70	474	7/19/2021	2.24	Y	N
CRSCF21	SCVI	0.08	F	J	27.9	371	6/8/2021	1.35	Y	Y
CRSCF21	SCVI	0.08	F	J	27.9	371	6/21/2021	0.07	N	Y
CRSCH21	SCVI	0.06	M	J	47.9	427	6/14/2021	0.19	N	Y
CRSCL19	CRSC	0.00	F	J	30.6	384	July 2019	0.18	Y	N
CRSCLL21	SCVI	0.08	F	J	24.8	337	7/22/2021	0.68	N	Y
CRSCM19	CRSC	0.00	M	A	231.4	694	8/5/2021	2.46	Y	N
CRSCMM21	CRSC	0.00	M	A	238.7	706	7/25/2021	2.48	Y	Y
CRSCP21	CRSC	0.00	M	A	230.7	673	6/27/2021	10.2	Y	N
CRSCQQ21	SCVI	0.11	F	A	357.3	760	8/2/2021	6.03	Y	N
CRSCS21	SCVI	0.08	F	J	77.3	493	7/5/2021	1.72	N	Y
CRSCV21	SCVI	0.10	F	J	34.6	386	7/7/2021	0.99	N	Y
CRSCWW21	CRSC	0.05	M	A	566.04	951	8/7/2021	12.9	Y	N
CRSCXX21	CRSC	0.05	F	J	69.7	494	8/9/2021	0.12	N	N
CRVIA20	CRVI	0.99	M	A	353.6	857	8/31/2021	1.09	N	Y
CRVIA21	CRVI	1.00	M	J	32.8	371	6/3/2021	0.27	Y	Y
CRVID20	CRVI	1.00	M	A	222.5	761	9/29/2021	Na	N	N
CRVIDD20	CRVI	1.00	F	J	51.4	407	8/13/2020	0.45	Y	Y
CRVIF20	CRVI	1.00	M	A	382.1	803	June 2019	5.17	Y	N
CRVIGG20	CRVI	1.00	M	A	504.8	888	9/5/2020	5.14	N	Y
CRVIH21	CRVI	1.00	M	A	319.8	815	7/6/2021	0.77	N	N
CRVIHH20	CRVI	Na	M	A	264.1	683	9/5/2020	1.11	N	N
CRVIK20	CRVI	1.00	M	A	439.2	804	6/17/2020	0.50	Y	N
CRVIKK20	CRVI	1.00	M	A	180.7	638	9/4/2020	0.92	Y	N
CRVIL20	CRVI	1.00	M	A	342.3	796	6/19/2020	2.95	Y	N
CRVIL20	CRVI	1.00	M	A	371.5	795	9/9/2021	1.61	Y	N
CRVIL20	CRVI	1.00	M	A	371.5	795	10/29/2021	Na	N	N
CRVIM21	CRVI	1.00	M	J	33.9	347	7/29/2021	0.11	Y	N
CRVIO20	CRVI	1.00	M	A	230.3	716	6/27/2020	0.12	N	Y
CRVIP20	CRVI	1.00	M	A	315.6	816	5/22/2021	1.56	N	N
CRVIR20	CRVI	1.00	M	J	56.9	458	7/8/2020	1.10	Y	N
CRVIT20	CRVI	1.00	M	A	358.7	748	7/11/2020	1.58	Y	N
SCVID21	SCVI	0.45	M	A	213	643	9/16/2021	4.84	Y	N
SCVIDD19	SCVI	0.06	F	A	161.4	654	8/6/2019	0.95	Y	N
SCVIE20	SCVI	Na	M	J	68	461	6/22/2020	0.34	N	Y
SCVIE21	SCVI	0.44	M	A	331.1	783	7/30/2021	3.88	Y	N
SCVIEE19	SCVI	0.94	M	A	221.3	745	August 2019	0.61	Y	N
SCVIF20	SCVI	0.40	M	A	140.3	599	6/26/2020	1.15	Y	N
SCVIFF19	SCVI	0.87	M	A	174.4	634	8/6/2019	3.47	Y	N
SCVIL19	CRVI	0.98	M	A	192.8	710	8/28/2019	3.23	Y	N
SCVILL19	SCVI	0.81	F	A	209.3	667	8/22/2019	1.24	N	Y
SCVIN20	SCVI	0.81	M	A	194.5	686	7/24/2020	2.74	Y	N
SCVINN19	CRVI	0.98	M	A	368.62	829	6/23/2020	3.84	Y	N
SCVIP19	SCVI	0.56	M	A	322.7	763	June 2019	4.62	Y	N
SCVIP20	CRVI	1.00	M	A	385.5	778	9/1/2021	4.17	Y	N
SCVIS19	CRVI	0.96	M	J	55.9	445.6	June 2019	0.31	N	Y
SCVIT19	SCVI	0.82	M	A	146.5	610	July 2019	1.38	Y	N
SCVITT19	SCVI	0.43	F	J	12.9	242	8/24/2019	0.10	N	Y
SCVIU19	SCVI	0.16	M	J	51.9	422	July 2019	0.85	Y	N
SCVIUU19	SCVI	0.16	F	A	229.4	732	8/29/2019	2.84	Y	N
SCVIX19	SCVI	0.20	M	A	290.4	793	8/21/2019	0.23	N	N
SCVIY19	SCVI	0.60	M	J	57.2	443	July 2019	1.98	Y	Y

*Note*: The first four characters of Snake ID indicate the site where the snake was initially captured: CRSC, Mojave rattlesnake site (SW of the hybrid zone); CRVI, prairie rattlesnake site (NE of the hybrid zone); SCVI, within the hybrid zone; the following characters indicate ascension order and year (e.g., A21, first snake captured in 2021; AA21, 27th snake captured in 2021). HI, hybrid index. All females were not pregnant. Age: A, adult; J, juveniles. Masses are in grams. SVL, Snout–vent length of the snake in mm. Mammal remains were any combination of pelage, vibrissae, and teeth. Lizard remains were scales.

**TABLE A2:** Stomach content data.

Snake ID	Genetic group	HI	Sex	Age	Snake mass	SVL	Date palpated	Content
CRSCEE21	CRSC	0.05	F	J	70	474	7/17/2021	Merriam's kangaroo rat (*Dipodomys merriami*)
CRSCFF21	SCVI	0.21	F	J	60.1	443	7/18/2021	Unknown rodent
CRSCL19	CRSC	0.00	F	J	30.6	384	7/27/2019	Whiptail lizard (*Aspidoscelis* spp.)
CRVIJ20	CRVI	1.00	M	A	325.8	772	6/16/2020	Either Merriam's or Ord's kangaroo rat (*Dipodomys merriami* or *D. ordii*)
SCVIA21	SCVI	0.54	M	J	116.8	552	7/7/2021	Pocket mouse (*Chaetodipus* spp.)
SCVID19	SCVI	0.61	M	A	168.5	659	5/28/2019	Round‐tailed horned lizard (*Phrynosoma modestum*)
SCVIE21	SCVI	0.44	M	A	331.1	783	7/31/2021	Ord's kangaroo rat (*Dipodomys ordii*)

*Note*: The first four characters of Snake ID indicate the site where the snake was initially captured: CRSC, Mojave rattlesnake site (SW of the hybrid zone); CRVI, prairie rattlesnake site (NE of the hybrid zone); SCVI, within the hybrid zone; the following characters indicate ascension order and year (e.g., A21, first snake captured in 2021; AA21, 27th snake captured in 2021). HI, hybrid index. All females were not pregnant. Age: A, adult; J, juveniles. Masses are in grams. SVL, Snout–vent length of the snake in mm.
